# Innovations in TAVR: The Latest in Device Technology

**DOI:** 10.3390/jcm14144906

**Published:** 2025-07-10

**Authors:** Omar Sheikh, Errol Moras, Lorraine Mascarenhas, Sahar Samimi, Waleed T. Kayani, Syed Zaid

**Affiliations:** 1 Section of Cardiology, Baylor College of Medicine, Houston, TX 77030, USA; omar.sheikh@bcm.edu (O.S.); lorraine.mascarenhas@bcm.edu (L.M.); sahar.samimi@bcm.edu (S.S.); kayani@bcm.edu (W.T.K.); 2 Brigham and Women’s Hospital, Harvard Medical School, Boston, MA 02115, USA

**Keywords:** TAVR, THV design, leaflet modification

## Abstract

Aortic stenosis is the most prevalent valvular disease globally. Transcatheter aortic valve replacement (TAVR) has become a well-established treatment for aortic stenosis, offering outcomes comparable to surgical aortic valve replacement (SAVR). Its use has expanded to include younger, lower-risk patients and those with more complex anatomies. Recent advancements in TAVR include the increased adoption of transfemoral access, prosthesis designs optimized for challenging anatomies, enhanced delivery systems with repositioning capabilities, and outer skirts to minimize paravalvular leaks. Despite these innovations, several challenges remain. This review highlights recent updates in transcatheter heart valve (THV) systems, leaflet modification devices, and the current limitations of TAVR.

## 1. Introduction

The field of transcatheter aortic valve replacement (TAVR) has undergone remarkable advancements over the past decade, transitioning from a novel intervention for high-risk surgical candidates to a widely accepted treatment for aortic stenosis across diverse patient populations [1]. Technological progress has broadened the array of devices and techniques, providing new options for patients with complex anatomies and those with aortic regurgitation. However, these advancements bring challenges, particularly in ensuring the long-term durability of transcatheter valves and addressing the unique needs of younger patients who may face multiple interventions over their lifetime [2]. This review highlights the latest developments in TAVR technology, with a focus on novel transcatheter heart valve (THV) systems, innovative leaflet modification devices, and strategies to address current limitations, while exploring their implications for the lifetime management of valvular heart disease.

## 2. Novel THV Systems

### 2.1. Siegel THV System

The Siegel THV (MiRus LCC, Marietta, GA, USA) is a balloon-expandable valve (BEV) designed for transfemoral delivery using an ultra-low-profile 8 Fr expandable sheath, enabling access in patients with small or diseased iliofemoral vessels. It accommodates annular sizes from 19 to 27 mm and features a cylindrical rhenium frame with no foreshortening during deployment, aiding in precise positioning. The valve incorporates nickel-free dry porcine leaflets, a conformable skirt to reduce paravalvular leak, and commissural alignment flags for accurate orientation. Its nitride oxide coating reduces pannus formation and enhances durability.

**Figure 1:**
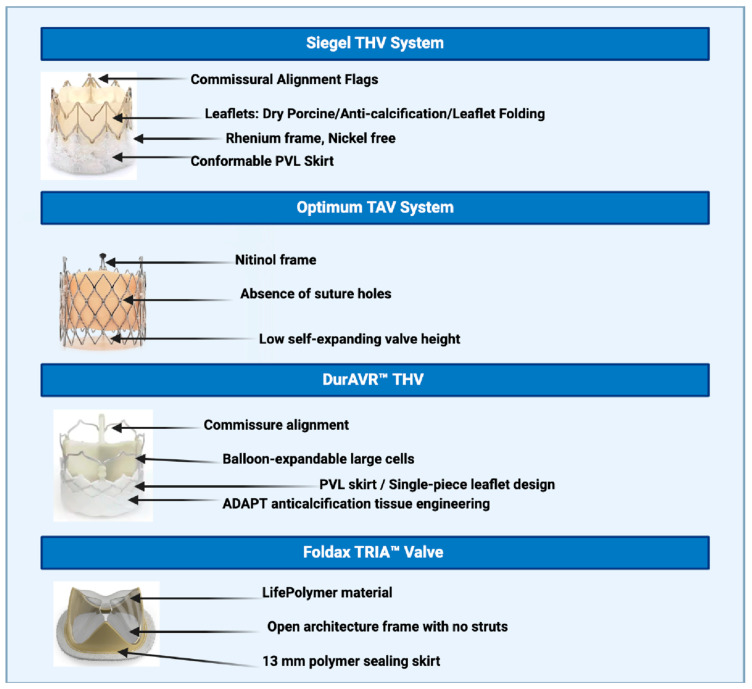
Overview of transcatheter heart valve systems showcasing unique structural components.

The Siegel THV system represents a notable advancement in THV technology, incorporating an innovative rhenium frame and nickel-free dry porcine leaflets designed to enhance durability and biocompatibility (Figure 1). The rhenium superalloy offers 2–3 times greater strength than traditional materials, reducing recoil and improving radiographic visibility. The conformable skirt ensures a superior seal to minimize PVL, while commissural alignment flags facilitate precise deployment, reducing the risk of misalignment. The nickel-free leaflets are particularly beneficial for patients with nickel allergies, mitigating the risk of allergic reactions [3,4]. Initial procedural outcomes have been encouraging, with first-in-human studies reporting successful deployment in patients with severe aortic stenosis and zero mortality or stroke at 30 days. The system demonstrated excellent hemodynamic performance, including reduced mean gradients, increased aortic valve area, with no requirement for a second valve or major vascular complications, highlighting its high success rate and minimal adverse events [5]. Compared with currently available balloon-expandable valves, the Siegel THV offers a lower frame height for improved coronary re-access and commissural alignment, alongside a tall skirt to minimize PVL, addressing the key limitations of prior-generation devices.

### 2.2. Optimum THV System

The Optimum TAV system (Thubrikar Aortic Valve, Collegeville, PA, USA) is a short, self-expanding platform with optimized geometry designed to enhance durability and leaflet dynamics (Figure 1). It features bovine pericardial tissue leaflets and a nitinol frame, which are both treated to minimize flexion stress and prevent calcification. It is delivered via transfemoral access using an 18 Fr system and is repositionable and retrievable. The valve is currently available for small annulus sizes (21–23 mm). With the lowest self-expanding valve height currently available, the system allows full coronary access post-implantation, reduces the risk of conduction abnormalities, and supports future valve-in-valve procedures. Its three leaflets, constructed from a single piece of tissue, ensure uniformity and durability. The absence of suture holes in mobile portions, particularly in the flexion zones prone to damage, further enhances durability. A tall porcine pericardial skirt mitigates risk of PVL through increased pliability, enabling better sealing behind the leaflets rather than below them. The system’s self-axial alignment capability reorients the valve during deployment for precise alignment with native leaflets. Additionally, it is fully repositionable and retrievable after partial deployment, ensuring accurate placement with minimal risk [6,7]. Early clinical results have demonstrated excellent valve gradients, effective orifice areas (EOA), and no moderate or severe aortic regurgitation. Patient outcomes remained favorable up to two years post-implantation, with only one pacemaker placement required for a tri-fascicular block [8]. Unlike conventional self-expanding valves, the Optimum TAV’s shortened frame height and natural valve geometry enhance coronary access and reduce mechanical stress on leaflets. Its unique single-sheet leaflet design and absence of suture holes in flexion zones aim to improve long-term durability compared to both balloon-expandable and self-expanding commercial valves.

### 2.3. Evolut FX+ THV System

The Evolut FX+ (Medtronic, Minneapolis, MN, USA) is a self-expanding valve mounted on a nitinol frame, representing the latest evolution of the Evolut platform (Medtronic, Minneapolis, MN, USA). Designed to preserve the hemodynamic advantages of supra-annular leaflet positioning while addressing limitations in coronary re-access, FX+ introduces significant frame design enhancements. It is delivered via transfemoral access using the Evolut FX delivery catheter system, which includes an InLine sheath with 14 Fr equivalent profile for 23, 26, and 29 mm valves, and an 18 Fr equivalent profile for the 34 mm valve.

The Evolut FX+ is available in four sizes: 23 mm (for annuli 18–20 mm), 26 mm (20–23 mm), 29 mm (23–26 mm), and 34 mm (26–30 mm). The hallmark innovation of FX+ is the inclusion of three enlarged diamond-shaped cells spaced 120° apart to serve as dedicated coronary access windows. These windows allow for improved selective coronary engagement—even in cases of commissural misalignment or sinus sequestration—without compromising radial strength or anchoring.

Valve deployment is performed using the cusp overlap technique to optimize commissural alignment. In early clinical experience, selective coronary engagement was achieved within 30–120 s post-implantation, with imaging confirmation of ideal diamond alignment and uncompromised valve performance. This platform responds to the growing clinical need for re-accessible valves in younger patients or those with coronary artery disease, offering an important advancement in procedural planning for lifetime management. A summary of the structural features, delivery profiles, and distinguishing characteristics of the novel devices discussed above is provided in Table 1.

## 3. Novel Leaflet Technologies

### 3.1. DurAVR™ THV

The DurAVR™ THV (Anteris Technologies, Eagan, MN, USA) is designed to replicate the performance of a native aortic valve, featuring a native-shaped structure made from a single-piece bovine pericardial tissue treated with ADAPT tissue technology (Figure 1). This treatment incorporates biochemical processes that significantly reduce leaflet calcification, enhancing durability and hemodynamic performance. The single-tissue design, contoured to mimic the architecture of native aortic leaflets, enables larger effective orifice areas and improved flow patterns, as demonstrated by cardiac MRI. This design contrasts with conventional bioprosthetic valves, which typically consist of three separate leaflets sutured together, limiting full valve expansion [9]. Early first-in-human studies involving over 50 patients have shown consistent hemodynamic results, including sustained low gradients and large EOAs. The valve also demonstrated efficacy in valve-in-valve (ViV) procedures, achieving hemodynamics comparable to initial post-surgical results. DurAVR™ has shown promise in high-risk patients, particularly in those undergoing ViV interventions for degenerated surgical bioprostheses [10,11].

### 3.2. Foldax TRIA™ Valve

The TRIA™ valve (Foldax, Salt Lake City, UT, USA) incorporates a proprietary synthetic polymer designed to resist calcification and thrombus formation, offering potential for lifelong durability without compromising quality of life (Figure 1). By reducing reliance on animal-derived tissue, the valve represents a significant advancement in biocompatibility. Key engineering features include a non-flared anchor region that ensures consistent annular anchoring, minimizing the risk of left ventricular outflow tract migration; controlled-release locking tabs for predictable deployment; and an open architecture frame without struts between commissures, preserving coronary access. The hourglass-shaped frame conforms to native root anatomy by increasing the space between the frame and coronary ostia, while a 13 mm polymer sealing skirt effectively reduces paravalvular leak. Robotic manufacturing enhances precision and consistency, with the added potential for decentralized production [12]. Early clinical trials of the TRIA™ surgical and transcatheter valves have demonstrated excellent flow dynamics, minimal gradients, and no significant valve dysfunction or need for reintervention. In chronic ovine studies, the valve showed no visible adhesions, growth, or calcification at 90 days, underscoring its durability and biocompatibility [13].

### 3.3. BCI Polyphenol-Based Technology

Third-generation polymer valves leverage BCI’s polyphenol-based coating technology to address key limitations of traditional heart valves, including thrombogenicity, calcification, and mechanical degeneration. The polyphenol coating reduces thrombus formation by up to 60%, protein adhesion by up to 99%, and microorganism adhesion by up to 82% compared to untreated materials. This biostable, biocompatible material is engineered to withstand the mechanical stresses of heart valve function, offering significant potential for long-term durability. By incorporating polymer heart valves and eliminating xenogenic antigens, this technology reduces reliance on animal-derived tissue, minimizing associated immune responses [14]. Furthermore, polymer valves are amenable to efficient robotic manufacturing, enhancing scalability and availability. These attributes position polymer valves as a promising breakthrough for valve replacement, particularly in younger patients [15].

## 4. TAVR for Aortic Regurgitation

**Figure 2:**
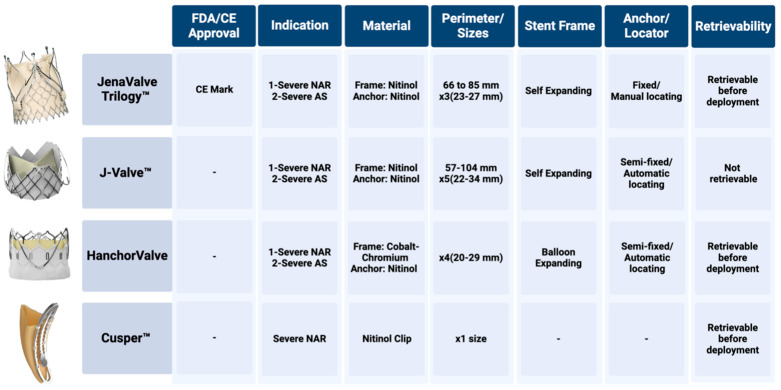
Representation of aortic regurgitation specific transcatheter devices.

Severe aortic regurgitation (AR) is a progressive condition with an annual mortality rate exceeding 10% if left untreated [16]. Despite the associated risks, nearly three-quarters of patients with severe symptomatic AR do not undergo surgical aortic valve replacement (SAVR) within the first year of diagnosis, primarily due to the high surgical risk [17,18]. TAVR is emerging as a viable alternative for patients with pure native AR, although it presents distinct technical challenges compared to aortic stenosis (AS). AR is often associated with a dilated annulus and aortic root with minimal calcification, compromising valve anchoring and stability [19,20]. Furthermore, the regurgitant jet, combined with enhanced left ventricular contractility, generates a suction effect that destabilizes the valve during implantation, complicating device control [19,20]. These anatomical and functional challenges have led to higher procedural complications and reintervention rates when using off-label TAVR devices originally designed for AS. The development of dedicated AR-specific devices, such as the JenaValve Trilogy™ (JenaValve Technology, Irvine, CA, USA), J-Valve™ (JC Medical, Burlingame, CA, USA), Hanchor valve (Healing Medical, Shanghai, China), and the Cusper™ device (Cuspa Medical, Nazareth, Israel), represents a significant advancement in addressing these limitations. The following sections will explore the design, hemodynamic performance, and clinical outcomes of these specialized devices (Figure 2).

### 4.1. JenaValve Trilogy™ System

The JenaValve Trilogy™ is a self-expanding valve (SEV) constructed from porcine pericardial tissue mounted on a nitinol frame with unique locators for native leaflet engagement, enabling use in aortic regurgitation without annular calcification. It is delivered transfemorally though an 18 Fr delivery system, and is available in sizes 23 mm, 25 mm, and 27 mm, accommodating annular perimeters from 66 to 85 mm.

The JenaValve Trilogy™ system marks a significant advancement in the transcatheter treatment of aortic valve diseases. It is the first transfemoral device to receive Conformité Européenne (CE) mark approval for treating both severe symptomatic AR and AS. Its key components include three radiopaque locators that attach to the native aortic leaflets, ensuring precise placement while limiting implant depth. These locators provide an anchoring mechanism and enhance the seal around the THV. The frame, with large open cells measuring 27–31 French (89–102 mm in diameter), allows coronary access after implantation and is fully retrievable and repositionable.

Clinically, the JenaValve Trilogy™ system has demonstrated excellent hemodynamic performance. Notably, 100% of patients achieved a mean pressure gradient below 20 mmHg at discharge and within 30 days post-procedure. In the ALIGN-AR trial, the system demonstrated a mean EOA of 2.9 cm^2^ and a mean gradient of 3.9 mmHg at 30 days, a performance comparable to TAVR devices used for AS [21]. In a large multicenter study by Adam et al., 90% of patients exhibited no or trace PVL at discharge, with a mean aortic valve gradient of 4.3 ± 1.6 mmHg and an average EOA of 2.7 ± 0.6 cm^2^. These hemodynamic outcomes remained stable at 30 days, with a mean gradient of 4.5 ± 2.0 mmHg [22].

Early European experiences and findings from the ALIGN-AR trial (NCT 04415047) underscore the system’s promising safety and efficacy profile in high-surgical-risk patients [21,22,23]. The locator technology is a key element responsible for the system’s significantly lower rates of valve embolization and migration, long considered the Achilles’ heel of TAVR in pure native AR. In a meta-analysis comparing outcomes of dedicated versus off-label devices for native AR, the pooled embolization/migration rate for THVs was 2%, with a permanent pacemaker implantation rate of 11%, compared to 8% and 20%, respectively, for off-label devices [24]. Additionally, the system achieves commissural alignment, which may further reduce the incidence of residual AR while maintaining adequate coronary access. The valve’s retrievability before final deployment adds an additional layer of safety, allowing for procedural adjustments.

### 4.2. J-Valve™

The J-Valve™ is a self-expanding valve (SEV) consisting of bovine pericardial leaflets attached to a nitinol stent frame, with three U-shaped anchor rings for supra-annular fixation. It is delivered via both transapical and transfemoral access, with transfemoral versions currently under investigation. It is available in five sizes—22 mm, 25 mm, 28 mm, 31 mm, and 34 mm, accommodating annular perimeters from 57 to 107 mm, and is compatible with an 18–21 Fr delivery system. It is CE-marked for transapical use, and is undergoing clinical evaluation for transfemoral deployment.

The J-Valve™ system is specifically designed to address the unique challenges of pure native AR without relying on calcification for anchoring. The alignment feature comprising three U-shaped Nitinol anchor graspers and large cells enables secure fixation within the aortic root while maintaining easy re-access to the coronary arteries. In an early feasibility study involving 15 patients with severe symptomatic AR, the J-Valve™ system demonstrated excellent hemodynamic performance, achieving a 30-day mean EOA of 2.9 ± 0.68 cm^2^, a transvalvular gradient of 5.5 ± 2.0 mmHg, and no or trace residual AR in all patients. Paired analysis at discharge and 30 days also showed evidence of left ventricular remodeling (NCT06034028) [25].

The system has demonstrated a high procedural success rate in both early feasibility and compassionate use studies, effectively reducing AR with minimal procedural complications. Importantly, it addresses an unmet clinical need by enabling treatment for patients with large aortic annuli, a population excluded from the ALIGN-AR trial. These early findings will inform the design of the upcoming JOURNEY pivotal trial [21].

### 4.3. Hanchor Valve

The Hanchor valve is a balloon-expandable valve (BEV) designed specifically for the treatment of pure native aortic regurgitation. It features a semi-fixed anchoring element for automatic sinus location and commissural alignment. The system is transfemorally delivered using an 18 Fr expandable sheath, with valve sizes of 20 mm, 23 mm, 26 mm, and 29 mm. The Hanchor valve represents a new paradigm in the TAVR landscape as the first balloon-expandable transfemoral TAVR system indicated for both pure native AR and AS. It features a cobalt–chromium alloy frame and bovine pericardial leaflets, along with a semi-fixed anchor element that ensures precise and secure positioning within the aortic root. The valve’s design incorporates automatic commissural alignment, simplifying the procedure and enhancing clinical outcomes [26]. Early clinical trials conducted in China have demonstrated a high procedural success rate with the Hanchor valve, showing significant improvements in hemodynamic parameters and no incidence of PVL [26]. The valve is currently under evaluation in the HAVE AR trial, a multicenter study aimed at confirming its safety and efficacy in high-risk patients with pure native AR.

### 4.4. Cusper™ Device

The Cusper™ device is a self-expanding transcatheter clip-based repair system, composed of a nitinol clip (3.4 mm × 16 mm) with an attached pericardial sac (6 mm diameter). It is delivered transfemorally via a 16 Fr dedicated delivery system with proximal and distal fixation holders for controlled release. The Cusper™ device introduces an innovative approach to transcatheter aortic valve repair in the management of severe AR. It employs a novel technique that is particularly advantageous in cases where traditional valve replacement is neither necessary nor feasible. The device consists of a nitinol clip and a pericardial sac that integrates seamlessly with the native aortic valve, preventing regurgitant blood flow into the left ventricle during diastole. The Cusper™ device is engineered to fill the effective regurgitant orifice of the aortic valve, while reducing the risk of thromboembolic events and preserving the potential for future valve replacement [27].

Currently in early clinical development, the Cusper™ device has demonstrated promising results in preclinical proof-of-concept studies, with a first-in-human study planned for 2025. Initial data suggest that the device effectively repairs aortic cusps while minimizing complications such as permanent pacemaker implantation, coronary occlusion, and thrombotic events. These findings indicate that Cusper™ could provide a flexible and transformative treatment option, particularly for patients who may require additional interventions later in life [27].

## 5. Leaflet Modification Technologies

**Figure 3:**
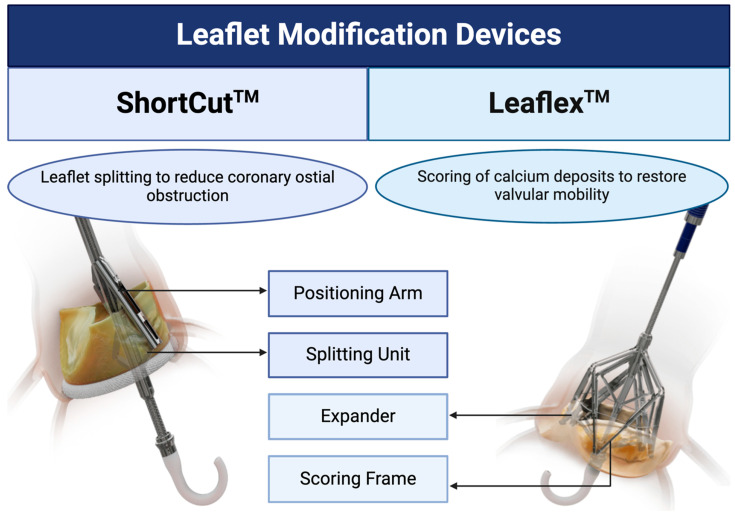
Leaflet technologies for transcatheter interventions.

Among the potential complications of TAVR, coronary artery obstruction is one of the most feared [28,29]. This risk is particularly pronounced in valve-in-valve (ViV) procedures, as the leaflets of the previously implanted valve may shift toward the coronary ostia, resulting in obstruction [30]. While certain anatomical features, such as low coronary height (<10 mm), narrow sinus of Valsalva (<30 mm), and a virtual valve-to-coronary artery distance of <4 mm, have been associated with this risk, predicting coronary obstruction remains challenging [31,32]. To address this issue in both native-valve and ViV TAVR procedures, leaflet modification techniques have emerged (Figure 3). Current strategies include coronary artery ostia protection using stenting techniques or electrocautery to lacerate the obstructing native or bioprosthetic leaflet [28,30]. Dedicated devices for leaflet modification are also being developed to tackle this technical challenge.

### 5.1. ShortCut™ Device

The ShortCut™ (Pi-Cardia, Rehovot, Israel) device is designed to mechanically split bioprosthetic valve leaflets, thereby reducing the risk of coronary obstruction. The device consists of a handle, a distal splitting unit that penetrates the leaflet in a controlled manner, and a positioning arm that safeguards the splitting unit and surrounding tissues throughout the procedure [29,33]. The ShortCut™ is introduced via a 16-Fr introducer sheath and advanced to the left ventricle before being unsheathed. Upon deployment, the positioning arm is rotated toward the targeted leaflet and coronary sinus, aligning with the respective coronary cusp. Once properly positioned, the splitting unit is deployed against the ventricular side of the leaflet, creating a controlled tear by locking the unit with the positioning arm on the opposite side of the leaflet and gently retracting it. After the process is complete, the splitting unit is re-engaged with the positioning arm, allowing the device to be rotated toward another leaflet if necessary [29,33].

Preliminary results from a pivotal trial demonstrated encouraging outcomes with the ShortCut™ in eight patients undergoing valve-in-valve (ViV) TAVR. The median procedural time was 19 min (IQR 16–23 min), with 100% 30-day survival and no reported cases of coronary obstruction or stroke at a median follow-up of 17 months [33]. Compared to traditional techniques, the ShortCut™ device offers an efficient, user-friendly approach to maintaining coronary ostial access during TAVR procedures.

### 5.2. Leaflex™ Device

While TAVR is increasingly used for patients with aortic stenosis, non-implant treatment options are emerging to address the underlying cause of calcific aortic stenosis [34]. For patients with multiple comorbidities and limited functionality, non-implant therapeutic interventions may offer significant benefits. The Leaflex™ (Pi-Cardia, Rehovot, Israel) is an innovative device that provides a treatment alternative for this cohort [35]. By scoring calcium deposits embedded in the valve leaflets, the Leaflex™ catheter restores leaflet mobility and improves valve function [34,35,36].

The Leaflex™ device consists of a frame with scoring elements and an expander that pushes the leaflets against the frame. Introduced via a 16 Fr introducer sheath, the expander is advanced past the aortic valve into the left ventricle, where it is unsheathed. The Frame is positioned proximally on the catheter, above the valve. Once the expander is retracted into the aortic valve, it expands to push the leaflets against the frame, creating two scoring lines per leaflet. If additional scoring is required, the frame can be repositioned, and the expander re-expanded to achieve optimal results [34,35,36].

In early feasibility studies, the Leaflex™ procedure significantly increased the aortic valve area and reduced mean pressure gradients [35]. Histological and micro-computed tomography evaluations demonstrated preserved tissue integrity on the ventricular surface of the leaflets. The Leaflex™ provides a novel alternative for managing aortic stenosis in patients seeking to avoid an implanted valve through TAVR or SAVR, or in those facing financial barriers to these more costly interventions.

## 6. Impact on Lifetime Management

With increasing life expectancy and a growing preference for bioprosthetic valves among younger and middle-aged patients, careful planning is essential to address the potential need for future interventions as patients outlive the durability of their initial valve [37]. The decision between SAVR and TAVR as the primary procedure requires a multidisciplinary heart team approach, factoring in patient preferences, age, surgical risk, comorbidities, anatomic considerations, and the implications for future procedures [2].

If TAVR is chosen, valve selection and implantation strategies should prioritize both immediate outcomes and the feasibility of subsequent TAV-in-TAV procedures [38]. Alternatively, opting for SAVR necessitates consideration of valve type and the potential need for aortic root enlargement to accommodate future valve-in-valve interventions [39]. Reintervention options for failed transcatheter THVs due to SVD include redo-TAVR, TAVR-explant, or medical management, with the choice dependent on clinical presentation, anatomic factors, and the mechanism of THV failure [40]. Challenges in TAV-in-TAV include neo-skirt height, prior THV expansion, leaflet overhang, and coronary access [41]. In cases of severe patient–prosthesis mismatch or endocarditis, TAVR-explant is often preferred, as redo-TAVR may not address these issues. Regular monitoring and advancements in imaging tools will play a critical role in detecting valve degeneration early and facilitating timely reintervention, improving the long-term management of these patients.

## 7. Unmet Needs and Knowledge Gaps

Balancing the competing risks of thrombosis and bleeding remains a persistent challenge in the post-TAVR phase. There is limited data on the optimal use of oral anticoagulants to prevent valve thrombosis in TAVR patients with high bleeding risk [42]. Minimizing thrombotic and hemorrhagic complications over the long term is a critical goal to optimize clinical outcomes. Despite reduced procedural complications and the expansion of TAVR to low-surgical-risk patients, evidence supporting the use of guideline-directed medical therapy for heart failure in TAVR patients is scarce, emphasizing the need for future clinical trials investigating left ventricular (LV) remodeling and recurrent HF events [43].

There is a pressing need for long-term studies spanning 15–20 years to provide comprehensive data on the durability of both TAVR and SAVR, particularly in younger patient populations. Current evidence is limited to a 10-year timeframe, leaving significant gaps in understanding the lifespan and performance of these devices over a patient’s lifetime. Predictive tools that assess a patient’s risk for future interventions, such as TAV-in-TAV or surgical explantation, based on individual anatomical and procedural factors, are urgently needed. Such tools would enable personalized treatment planning, supporting optimal initial intervention strategies and anticipating potential complications [44].

Current guidelines exhibit variability, particularly regarding TAVR use in younger and lower-risk patients. Standardized, evidence-based guidelines are necessary to ensure consistent and optimal patient care. The development of clear protocols for lifetime management, including criteria for re-intervention, would reduce variability in clinical practice. Innovative valve designs are needed to address the limitations of current devices, such as enhancing durability, minimizing the risk of coronary obstruction, and facilitating easier re-interventions [45]. Emerging technologies, including devices like ShortCut™ and Leaflex™, demonstrate promising potential to meet these unmet needs through novel materials and engineering approaches.

## 8. Evolving Patient Selection and Clinical Impact

The ongoing evolution in TAVR device design is likely to shift current paradigms in patient selection. While traditional TAVR candidates have been defined by symptomatic severe aortic stenosis with significant annular calcification, several next-generation platforms such as the JenaValve Trilogy™, J-Valve™, and Hanchor valve are being specifically developed to address the needs of patients with pure native aortic regurgitation, previously excluded from TAVR eligibility. Similarly, leaflet repair devices like Cusper™ introduce a novel paradigm where valve function can be restored without complete valve replacement, potentially benefiting younger patients with isolated leaflet dysfunction. Additionally, the low-profile access systems and commissural alignment capabilities of devices like the Siegel THV and Optimum TAV may improve procedural feasibility in patients with small iliofemoral vessels or horizontal aortas. These innovations collectively signal a future expansion of TAVR indications, both toward lower-risk populations and those with previously ineligible anatomies.

## 9. Future Directions

Aortic valve interventions have experienced exponential growth, drawing significant interest from all aspects of cardiovascular care, including medical device innovation. Despite advancements in device technology, the continued expansion of the percutaneous aortic field to younger and lower-risk populations, earlier intervention in asymptomatic patients and less severe aortic stenosis underscores the need for further innovation. Emerging technologies detailed in our review hold promise in improving the safety and efficacy of re-interventions and expanding treatment options for complex anatomies. Long-term clinical trials are crucial to generating the data necessary for refining guidelines and optimizing treatment strategies, especially for patients requiring multiple valve interventions throughout their lifetime. In particular, devices specifically designed for aortic regurgitation may catalyze future updates to guideline-directed therapy, expanding indications beyond calcific stenosis and establishing formal criteria for transcatheter treatment of pure AR. In parallel, pharmacologic strategies may complement device-based therapy in optimizing post-TAVR outcomes; for instance, sodium–glucose cotransporter-2 (SGLT2) inhibitors have been shown to enhance cardiac remodeling and long-term survival in diabetic patients undergoing TAVR [46]. As our understanding of structural–functional interplay deepens, such adjunctive therapies may emerge as important tools in promoting myocardial recovery and improving the durability of procedural success.

Shared decision-making will play an increasingly critical role in aligning treatment plans with patient values, preferences, and life goals. Educating patients about the risks and benefits of TAVR versus SAVR, along with the potential for future interventions, will be essential to achieving the best possible long-term outcomes. With the transition of these novel devices from feasibility studies to broader clinical use, economic sustainability will become a critical determinant of adoption. Variability in manufacturing complexity, durability, and procedural resources may lead to significant differences in device-associated costs. Future comparative studies should incorporate health economic analyses to evaluate cost-effectiveness, guide resource allocation, and inform clinical decision-making in diverse healthcare settings.

## 10. Conclusions

Recent advancements in TAVR technology, including novel THV systems and leaflet modification devices, have significantly enhanced the treatment of aortic stenosis and regurgitation. These innovations have improved procedural outcomes, reduced complications, and broadened the range of patients eligible for minimally invasive valve replacement. However, challenges persist in ensuring long-term valve durability, facilitating re-interventions, and managing complex anatomies, as well as younger and lower-risk patients who may require multiple valve procedures over their lifetime. The intricacies of lifetime management, particularly the risks associated with TAV-in-TAV procedures and the potential for surgical explantation, highlight the critical need for ongoing research and technological innovation.

## Figures and Tables

**Table 1:** Key characteristics of novel transcatheter heart valve systems and associated technologies.

Device Name	Leaflet Material	Frame Type	Valve Sizes	Delivery System	Special Features
**Novel THV Systems**					
Siegel	Nickel-free porcine	BEV, cobalt–chromium	Compatible with annular diameters 19–27 mm	8 Fr	Short frame, rhenium alloy, commissural alignment, tall sealing skirt, nickel-free leaflets
Optimum	Bovine pericardial	SEV, nitinol	25 mm	18 Fr	Shortest SEV height, single-sheet leaflet, commissural alignment, retrievable
Evolut FX+	Porcine pericardial	SEV, nitinol	23, 26, 29, 34 mm	14–18 Fr (InLine)	Enlarged coronary access cells, dot markers for commissural alignment, repositionable
**Novel Leaflet Technologies**					
DurAVR	Single-piece bovine pericardial	SEV, nitinol			ADAPT-treated tissue, native-shaped, no sutures, high EOA
Foldax TRIA	Synthetic polymer	Open architecture polymer frame			Robotic manufacturing, anti-calcification, maintains coronary access
BCI Polymer Valve	Polyphenol-coated polymer	Biostable polymer			Anti-thrombotic, low protein adhesion, no xenogenic tissue
**TAVR for Aortic Regurgitation**					
JenaValve Trilogy	Porcine pericardial	SEV, nitinol	23, 25, 27 mm	18 Fr	Locator technology, retrievable, CE-approved for AR and AS
J-Valve	Bovine pericardial	SEV, nitinol with graspers	22, 25, 28, 31, 34 mm	18–21 Fr	U-shaped anchor rings, LV remodeling, large annulus compatibility
Hanchor Valve	Bovine pericardial	BEV, cobalt–chromium	20, 23, 26, 29 mm	18 Fr	Semi-fixed anchor, automatic commissural alignment
Cusper	Pericardial sac (repair)	Self-expanding nitinol clip	1 size (clip)	16 Fr	Repairs cusps, preserves anatomy, non-replacement device
**Leaflet Modification Technologies**					
ShortCut	N/A	N/A (splitting device)		16 Fr	Mechanical leaflet splitting, for ViV coronary protection
Leaflex	Native leaflet scoring	N/A (non-implant scoring device)		16 Fr	Calcium scoring, improves mobility, non-implant therapy

AR = aortic regurgitation; AS = aortic stenosis; BEV = balloon-expandable valve; CE = Conformité Européenne; EOA = effective orifice area; Fr = French (catheter size); FX = frame extension; SEV = self-expanding valve; TAVR = transcatheter aortic valve replacement; THV = transcatheter heart valve; ViV = valve-in-valve; N/A = not applicable.

## References

[1] Davidson L.J., Davidson C.J. (2021). Transcatheter Treatment of Valvular Heart Disease: A Review. JAMA.

[2] Jubran A., Patel R.V., Sathananthan J., Wijeysundera H.C. (2024). Lifetime Management of Patients with Severe Aortic Stenosis in the Era of Transcatheter Aortic Valve Replacement. Can. J. Cardiol..

[3] SIEGEL™ Transcatheter Aortic Valve. MIRus. https://www.mirusmed.com/solutions/structural-heart/siegel-transcatheter-aortic-valve-2/.

[4] The Ballon Expandable Siegel TAVR System: A Low-Profile Molybdenum-Rhenium Alloy Frame Design (MIRus). https://www.tctmd.com/slide/ballon-expandable-siegel-tavr-system-low-profile-molybdenum-rhenium-alloy-frame-design-mirus.

[5] Cowart P. (2024). MiRus Siegel™ TAVR: First in Human Results Presented at New York Valves. MiRus. https://www.mirusmed.com/mirus-siegel-tavr-first-in-human-results-presented-at-new-york-valves/.

[6] Optimum TAVI System—Thubrikar Aortic Valve. https://tavi.us/optimum-tav/.

[7] The Optimum Short Frame Self-Expanding TAVR System (Optimum TAV). https://www.tctmd.com/slide/optimum-short-frame-self-expanding-tavr-system-optimum-tav.

[8] (2024). Thubrikar Marks Success of First Optimum TAV Implants Using its Precision 2 Catheter. Medical Product Outsourcing. https://www.mpo-mag.com/breaking-news/thubrikar-marks-success-of-first-optimum-tav-implants-using-its-precision-2-catheter/.

[9] DurAVR® THV Clinical Evidence|Anteris Technologies. https://anteristech.com/clinical-evidence.html.

[10] Kodali S.K., Sorajja P., Meduri C.U., Feldt K., Cavalcante J.L., Garg P., Hamid N., Poon K.K., Settergren M.R.M., Burns M.R. Early Safety and Feasibility of a First-In-Class Biomimetic Transcatheter Aortic Valve—DurAVR. https://eurointervention.pcronline.com/article/early-safety-and-feasibility-of-a-first-in-class-biomimetic-transcatheter-aortic-valve-duravr.

[11] Meduri C., Latib A., Kodali S., Feldt K., Garg P., Cavalcante J., Sorajja P., Hamid N., Sathananthan J., Poon K. (2023). TCT-467 DurAVR Biomimetic Transcatheter Aortic Valve: First-in-Human Study Results Update. J. Am. Coll. Cardiol..

[12] Foldax. https://www.foldax.com/.

[13] PCR TRIA™ Polymer Surgical Valves Surpass 200 Patient Life Years. https://www.pcronline.com/News/Press-releases/2024/TRIA-polymer-surgical-valves-surpass-200-patient-life-years.

[14] Bolaños-Cardet J., Ruiz-Molina D., Yuste V.J., Suárez-García S. (2024). Bioinspired phenol-based coatings for medical fabrics against antimicrobial resistance. Chem. Eng. J..

[15] A Cutting-Edge Polyphenol-Based Coating Technology for the Third Generation of Polymer Heart Valves (BCI Technology). https://www.tctmd.com/slide/cutting-edge-polyphenol-based-coating-technology-third-generation-polymer-heart-valves-bci.

[16] Bekeredjian R., Grayburn P.A. (2005). Valvular Heart Disease: Aortic Regurgitation. Circulation.

[17] Thourani V.H., Brennan J.M., Edelman J.J., Chen Q., Boero I.J., Sarkar R.R., Murphy S.M.E., Leon M.B., Kodali S.K. (2021). Treatment Patterns, Disparities, and Management Strategies Impact Clinical Outcomes in Patients with Symptomatic Severe Aortic Regurgitation. Struct. Heart.

[18] Iung B., Delgado V., Rosenhek R., Price S., Prendergast B., Wendler O., De Bonis M., Tribouilloy C., Evangelista A., Bogachev-Prokophiev A. (2019). Contemporary Presentation and Management of Valvular Heart Disease: The EURObservational Research Programme Valvular Heart Disease II Survey. Circulation.

[19] Poletti E., De Backer O., Scotti A., Costa G., Bruno F., Fiorina C., Buzzatti N., Latini A., Rudolph T.K., Van Den Dorpel M.M.P. (2023). Transcatheter Aortic Valve Replacement for Pure Native Aortic Valve Regurgitation. JACC Cardiovasc. Interv..

[20] De Backer O., Pilgrim T., Simonato M., Mackensen G.B., Fiorina C., Veulemanns V., Cerillo A., Schofer J., Amabile N., Achkouty G. (2018). Usefulness of Transcatheter Aortic Valve Implantation for Treatment of Pure Native Aortic Valve Regurgitation. Am. J. Cardiol..

[21] Vahl T.P., Thourani V.H., Makkar R.R., Hamid N., Khalique O.K., Daniels D., McCabe J.M., Satler L., Russo M., Cheng W. (2024). Transcatheter aortic valve implantation in patients with high-risk symptomatic native aortic regurgitation (ALIGN-AR): A prospective, multicentre, single-arm study. Lancet.

[22] Adam M., Tamm A.R., Wienemann H., Unbehaun A., Klein C., Arnold M., Marwan M., Theiss H., Braun D., Bleiziffer S. (2023). Transcatheter Aortic Valve Replacement for Isolated Aortic Regurgitation Using a New Self-Expanding TAVR System. JACC Cardiovasc. Interv..

[23] Poletti E., Adam M., Wienemann H., Sisinni A., Patel K.P., Amat-Santos I.J., Orzalkiewicz M., Saia F., Regazzoli D., Fiorina C. (2024). Performance of Purpose-Built vs Off-Label Transcatheter Devices for Aortic Regurgitation. JACC Cardiovasc. Interv..

[24] Samimi S., Hatab T., Kharsa C., Khan S., Bou Chaaya R., Qamar F., Aoun J., Zaid S., Faza N., Atkins M.D. (2025). Meta-analysis of Dedicated Versus Off-label Transcatheter Devices for Native Aortic Regurgitation. Cardiovasc. Interv..

[25] Garcia S., Kaneko T., Reardon M., Goel S., Cohen D.J., Cavalcante J.L., Chuang M.L., Hahn R.T., Latib A., Waksman R. (2024). Treatment of Aortic Regurgitation with a Novel Device. JACC Cardiovasc. Interv..

[26] Pan W. A Novel Transfemoral Aortic Valve Replacement System (Hanchor Valve) for Pure Native Aortic Regurgitation (PNAR). [Conference Presentation Slides]. Transcatheter Cardiovascular Therapeutics. October 2024. https://www.crfconnect.com/episode/a-novel-transfemoral-aortic-valve-replacement-system-hanchor-valve-for-pure-native-aortic-regurgitation-pnar-1653.

[27] Feld Y., Yakobi D., Hazan S., Weigler A. (2022). Transcatheter aortic valve repair for aortic regurgitation with the Cusper device. EuroIntervention.

[28] Haberman D., Chitturi K.R., Waksman R. (2024). Leaflet modification with the ShortCut™ device to prevent coronary artery obstruction during TAVR. Cardiovasc. Revascularization Med. Mol. Interv..

[29] Tchétché D., Kodali S.K., Dvir D. First Dedicated Transcatheter Leaflet Splitting Device: The ShortCut Device. https://eurointervention.pcronline.com/article/first-dedicated-transcatheter-leaflet-splitting-device-the-shortcut-device.

[30] Dvir D., Tchétché D., Leon M.B., Généreux P., Seguy B., Makkar R., Pibarot P., Gada H., Nazif T., Hildick-Smith D. (2024). Leaflet modification before transcatheter aortic valve implantation in patients at risk for coronary obstruction: The ShortCut study. Eur. Heart J..

[31] Ojeda S., González-Manzanares R., Jiménez-Quevedo P., Piñón P., Asmarats L., Amat-Santos I., Fernández-Nofrerias E., Valle R.D., Muñoz-García E., Ferrer-Gracia M.-C. (2023). Coronary Obstruction After Transcatheter Aortic Valve Replacement: Insights From the Spanish TAVI Registry. JACC Cardiovasc. Interv..

[32] Blanke P., Soon J., Dvir D., Park J.K., Naoum C., Kueh S.-H., Wood D.A., Norgaard B.L., Selvakumar K., Ye J. (2016). Computed tomography assessment for transcatheter aortic valve in valve implantation: The vancouver approach to predict anatomical risk for coronary obstruction and other considerations. J. Cardiovasc. Comput. Tomogr..

[33] Dvir D., Leon M.B., Abdel-Wahab M., Unbehaun A., Kodali S., Tchetche D., Pibarot P., Leipsic J., Blanke P., Gerckens U. (2023). First-in-Human Dedicated Leaflet Splitting Device for Prevention of Coronary Obstruction in Transcatheter Aortic Valve Replacement. JACC Cardiovasc. Interv..

[34] Baumbach A., Mylotte D., Hildick-Smith D., Kennon S., Jonas M., Bartus K., Trebacz J., Halevi R., Kislev Y., Plotnikov L. (2021). Non-implant valve repair for calcific aortic stenosis: The Leaflex study. EuroIntervention J. Eur. Collab. Work. Group Interv. Cardiol. Eur. Soc. Cardiol..

[35] Bartus K., Surve D., Sato Y., Halevi R., Kislev Y., Sax S., Markov L., Golan E., Levy R., Halon D. (2020). The Leaflex™ Catheter—A Novel Device for Treating Calcific Aortic Stenosis—First-in-Human Intra-Operative Assessment of Safety and Efficacy. Struct. Heart.

[36] Jonas M., Rozenman Y., Moshkovitz Y., Hamdan A., Kislev Y., Tirosh N., Sax S., Trumer D., Golan E., Raanani E. The Leaflex™ Catheter System—A Viable Treatment Option Alongside Valve Replacement? Preclinical Feasibility of a Novel Device Designed for Fracturing Aortic Valve Calcification. https://eurointervention.pcronline.com/article/the-leaflex-catheter-system-a-viable-treatment-option-alongside-valve-replacement-preclinical-feasibility-of-a-novel-device-designed-for-fracturing-aortic-valve-calcification.

[37] Russo G., Tang G.H.L., Sangiorgi G., Pedicino D., Enriquez-Sarano M., Maisano F., Taramasso M. (2022). Lifetime Management of Aortic Stenosis: Transcatheter Versus Surgical Treatment for Young and Low-Risk Patients. Circ. Cardiovasc. Interv..

[38] Bapat V.N., Zaid S., Fukuhara S., Saha S., Vitanova K., Kiefer P., Squiers J.J., Voisine P., Pirelli L., von Ballmoos M.W. (2021). Surgical Explantation After TAVR Failure: Mid-Term Outcomes From the EXPLANT-TAVR International Registry. JACC Cardiovasc. Interv..

[39] Windecker S., Okuno T., Unbehaun A., Mack M., Kapadia S., Falk V. (2022). Which patients with aortic stenosis should be referred to surgery rather than transcatheter aortic valve implantation?. Eur. Heart J..

[40] Sá M.P.B.O., Van den Eynde J., Simonato M., Cavalcanti L.R.P., Doulamis I.P., Weixler V., Kampaktsis P.N., Gallo M., Laforgia P.L., Zhigalov K. (2021). Valve-in-Valve Transcatheter Aortic Valve Replacement Versus Redo Surgical Aortic Valve Replacement: An Updated Meta-Analysis. JACC Cardiovasc. Interv..

[41] Akodad M., Sellers S., Landes U., Meier D., Tang G.H.L., Gada H., Rogers T., Caskey M., Rutkin B., Puri R. (2022). Balloon-Expandable Valve for Treatment of Evolut Valve Failure. JACC Cardiovasc. Interv..

[42] Ng A.C.T., Holmes D.R., Mack M.J., Delgado V., Makkar R., Blanke P., Leipsic J.A., Leon M.B., Bax J.J. (2020). Leaflet immobility and thrombosis in transcatheter aortic valve replacement. Eur. Heart J..

[43] Valvo R., Costa G., Tamburino C., Barbanti M. (2019). Antithrombotic Therapy in Transcatheter Aortic Valve Replacement. Front. Cardiovasc. Med..

[44] Hayek A., Prieur C., Dürrleman N., Chatelain Q., Ibrahim R., Asgar A., Modine T., Ali W.B. (2024). Clinical considerations and challenges in TAV-in-TAV procedures. Front. Cardiovasc. Med..

[45] Zaid S., Bapat V.N., Sathananthan J., Landes U., De Backer O., Tarantini G., Grubb K.J., Kaneko T., Khalique O.K., Jilaihawi H. (2023). Challenges and Future Directions in Redo Aortic Valve Reintervention After Transcatheter Aortic Valve Replacement Failure. Circ. Cardiovasc. Interv..

[46] Paolisso P., Belmonte M., Gallinoro E., Scarsini R., Bergamaschi L., Portolan L., Armillotta M., Esposito G., Moscarella E., Benfari G. (2024). SGLT2-inhibitors in diabetic patients with severe aortic stenosis and cardiac damage undergoing transcatheter aortic valve implantation (TAVI). Cardiovasc. Diabetol..

